# Polish adaptation of the Family Resilience Assessment Scale (FRAS)

**DOI:** 10.1007/s10597-020-00626-3

**Published:** 2020-05-06

**Authors:** Natalia Nadrowska, Magdalena Błażek, Aleksandra Lewandowska-Walter

**Affiliations:** 1 Department of Quality of Life Research, Department of Psychology, Medical University of Gdańsk, 15 Tuwima Str, 80-210 Gdańsk, Poland; 2 Division of Research On Family and Quality of Life, Institute of Psychology, University of Gdańsk, Gdańsk, Poland

**Keywords:** Resilience, Family relations, Life changes events, Adaptation

## Abstract

Family Assessment Resilience Scale (FRAS) by Sixbey (2005) is based on the model proposed by Walsh (1996), allows to evaluate the processes of family resilience. The main goal of this study was to adapt the English version of FRAS to the Polish population (FRAS-PL) as there is no questionnaire in Poland to assess family resilience. After the process of translation into Polish and then back to English to check the correctness, the final version was accepted and participants (*N* = 502, 65% female, M = 24.60, SD = 6.86) filled out the questionnaire. To obtain the best-fitting model of the tool, confirmatory factor analysis (CFA) was used. Confirmatory Factor Analysis showed that the six-factor model fits the obtained data (χ^2^/df = 2.95, RMSEA = 0.06, CFI = 0.92, TLI = 0.91, WRMR = 1.87). The alpha coefficients are satisfactory for all subscales (from α = 0.63 to α = 0.95). The adaptation of FRAS-PL was compared to final versions accepted in different countries and explained in the cultural context. The Polish version of the scale, named FRAS-PL, can be used for both researchers and clinicians to assess family resilience.

## Introduction

Families are constantly experiencing situations that disrupt their balance. These situations are related to changes in family life routine and adaptation to them (Walsh 2013). The construct of family resilience allows researchers and practitioners to consider the family as a source of strength, and moves the attention from the individual family members to the whole family seen as a unit (Van Breda 2001). In this project family is understood as self-defined unit (not biological) as Walsh (1996) and Sixbey (2005) proposed. Modern families are diverse and experience many difficulties. This is why a multifaceted approach to family resilience is required. Such a contemporary approach to family resilience is represented by Walsh (1996, 2003, 2006, 2013) who also points out that the construct of family resilience must be considered as a long-term process, because families may seem overwhelmed by problems at first and then overcome them. According to the literature, factors such as individual family response to stress, life cycle of the family, type of stressor or cultural context should be taken into account in the assessment of the family resilience (DeHaan et al. 2013; Walsh 2006).

Walsh (2006) defined family resilience as the path that families follow in response to stress. This concept refers to both ways of coping with stress and adaptation to a stressful situation (to what a family does to survive a difficult time and to what enables it to adapt), this also refers to acquiring new resources and developing family potential in response to stress (change of family priorities, what family gain, what family learn). Generally, family resilience refers to the processes taking place in the family and all types of activities undertaken by the family members, which enable it not only to survive a difficult time but also to become stronger after the crisis (Walsh 2006, 2013). Creating the family resilience construct Walsh based on her clinical work (see: Walsh 1978, 2014), as well as on a family systemic approach (see: Walsh 1996; Patterson 2002), both ecological (see: Falicov 1988, 1995) and developmental (see: Rolland 1987; Walsh 2013). This concept takes into account the constant changes in the family and refers to the time of crisis, the time before and after the crisis. It enables assessment of different family structures (e.g. single parent’s family, blended family), different types of crisis (family member's illness, traffic accident, sudden death), and also refers to family coping with everyday stressors (Walsh 2006).

Walsh (1996, 2003, 2006, 2013) posits that the processes of family resilience are related to belief systems, family organizational patterns, communication and problem solving. These processes help to cope with changes, stress, predictable (normative) and unpredictable (non-normative) crises, as well as adaptation to changing living conditions, through increasing the capacity of the family members, their relationships and the whole family. Each of the main family resilience processes is composed of three sub-processes. These processes are complementary and interactive. The first of the major processes is the *belief systems* and it consists of assigning a meaning to adversity (family members treat the crisis as a common challenge, thus stabilize the situation), *positive outlook* (family members focus on their potential, optimism, they believe in overcoming adversity) and *transcendence and spirituality* (family members seek strength in faith, seek contact with nature, get involved in social activities). The second is *organizational patterns of family* and it includes: *flexibility* (adaptive family changes, family reunification, strong family leadership), *connectedness* (mutual support, commitment), *social and economic resources* (support from relatives or institutions, financial security). The third is *communication and problem solving* and it consists of the following processes: *communication clarity* (family members seek the truth and seek clarification of ambiguous information, their communication is clear and coherent), *open emotional expression* (family members share feelings, engage in positive interactions, they spend time together) and *collaborative problem solving* (creativity, collaborative decision making, proactive attitude) (Walsh 2006).

Based on the Family Resilience Model by Walsh (1996, 2003, 2006, 2013), Sixbey (2005) developed a tool to test family resilience—*Family Resilience Assessment Scale* (FRAS). The questionnaire consists of subscales for assessing the areas of family resilience. These scales are part of the overall *Family Resilience* and this overarching factor refers to the general level of family resilience manifested by the family. *Family Communication and Problem Solving* (FCPS) subscale refers to sharing of information and feelings in a clear and open way when solving problems. *The Utilizing Social and Economic Resources* (USER) subscale addresses the issues of internal and external standards which enable the fulfillment of daily tasks by identifying useful resources. *The Maintaining a Positive Outlook* (MPO) subscale concerns hope expressed by the family to overcome adversity and to survive the difficult time seizing the opportunities. The subscale of *Family Connectedness* (FC) concerns mutual bonds while respecting the differences of each family member. The *Family Spirituality* subscale (FS) concerns the guiding system, that gives life a meaning and sense. The subscale of *Ability to Make Meaning of Adversity* (AMMA) defines the family's perceived difficult events as part of life.

The adaptation of the English version of FRAS was carried out in Turkey (Kaya and Neslihan 2012), Romania (Bostan 2014), Malta (Dimech 2014), China (Li et al. 2016) Croatia (Ferić et al. 2016) and South Africa (Isaacs et al. 2018). The versions of the scales in those countries differ in two aspects: the number of factors obtained in the analyzes and the presence of a total scale score. In some cases, the names of the subscales were changed due to cultural differences. In Turkey (Kaya and Neslihan 2012) the goodness of fit index values for the model were: χ^2^/df = 2.18, RMSEA = 0.058, CFI = 0.93, GFI = 0.92, IFI = 0.92, AGFI = 0.93, SRMR = 0.066 and a four-factor solution with 44 items was accepted. The following subscales were kept: *Family Communication and Problem Solving, Utilizing Social and Economic Resources, Maintaining a Positive Outlook, Ability to Make Meaning of Adversity*. In Romania (Bostan 2014), a six-factor solution was obtained, and the scales were maintained as in the original version of FRAS. The internal consistence coefficients for the Romanian version (Bostan 2014) were greater than 0.70 for three of the dimensions and for the other three lower than 0.70 (*Maintaining the Positive Outlook and Family Connectedness, Ability to Make Meaning of Adversity*). In Malta (Dimech 2014) the adaptation yielded a six-factor solution and three of the scales in this version obtained Cronbach Alpha above 0.70. In China (Li et al. 2016) the goodness of fit index values for the model were: χ^2^/df = 5.97, RMSEA = 0.08, CFI = 0.84, SRMR = 0.05 and a three-factor solution with 32 items was accepted. The following subscales were kept: *Family Communication and Problem Solving, Utilizing Social Resources, Maintaining a Positive Outlook*. Six-factor solution was obtained in the Croatian adaptation (Ferić et al. 2016), with the following scales: *Family Communication and Problem Solving, Making Sense of Adversity, Neighbors Support, Family Spirituality, Family Connection, Security and Support in the Community.* The Cronbach Alpha for Croatian version varied from 0.65–0.92. In South Africa (Isaacs et al. 2018) six factor solution was obtained, and it was very similar to that shown by Sixbey (2005) with Cronbach Alpha from 0.75 for Ability to Make Meaning of Adversity to 0.97 for Family Communication and Problem Solving (only Family Connectedness received lower Cronbach Alpha 0.38). The Cronbach’s alpha coefficient values for the total scale score were received in four countries with the value of 0.97 for the South African version, 0.95 for the Chinese version, 0.92 for the Turkish version, and 0.86 for the Maltese one.

There are also other scales used to assess family strength and family resilience such as Family Inventory of Resources for Management (FIRM), Family Celebrations Index (FCELEBI), Family Coping Coherence Index (FCCI), Social Support Index (SSI) and others (McCubbin et al. 1996). However, these scales do not reflect the overall construct of family resilience but rather its different aspects. Thus, we decided to adapt the tool to assess family resilience in Poland, because there is a gap in this area and the construct of family resilience is sporadically discussed in Polish literature (Gąsior 2012, 2014; Lachowska 2012, 2014). We already presented the results of research carried out on the Polish population with an experimental version of the questionnaire. Preliminary results are satisfying (hidden information, names of authors of the publication). For the purpose of our work we have chosen to adapt the tool, which combines the various aspect of family resilience. Family Resilience Assessment Scale (Sixbey 2005) covers and allows to evaluate the three main aspects of the family resilience construct: the belief system, the patterns of family organization, and communication as well as problem solving.

## Methods

### Questionnaire of FRAS

The Family Resilience Assessment Scale (FRAS) by Sixbey (2005) allows to assess the family functioning in terms of family resilience. Cronbach’s alpha for this entire scale (54 items) was 0.96 and creates the overarching factor of Family Resilience. The scale consists of six subscales: Family Communication and Problem Solving (FCPS, *α* = 0.96, with 27 items, e.g. *We are able to work through pain and come to an understanding* and *We discuss problems and feel good about the solutions*), Utilizing Social and Economic Resources (USER, *α* = 0.85, with eight items, e.g. *We know we are important to our friends* and *We think this is a good community to raise children*), Maintaining a Positive Outlook (MPO, *α* = 0.86, with six items, e.g. *We have the strength to solve our problems*), Family Connectedness (FC, *α* = 0.70, with 6 items, e.g. *Our friends value us and who we are*), Family Spirituality (FS, *α* = 0.88, with four items, e.g. *We participate in church activities*), Ability to Make Meaning of Adversity (AMMA, *α* = 0.96, with three items, e.g. *We accept that problems occur unexpectedly*). The participants evaluate each item on a 4-point scale (1—*strongly disagree* to 4—*strongly agree*). Four questions (items: 33, 37, 45, 50) need to be reversed. The higher the result, the higher the level of family resilience. Family Resilience Assessment Scale (FRAS) has good concurrent criterion validity with three scales: *Family Assessment Device 1* (FAD 1; *r* = 0.91) (Epstein et al. 1983), *Family Assessment Device 2* (FAD 2; *r* = 0.85) (Epstein et al. 1983), *Personal Meaning Index* (PMI; *r* = 0.85) (Reker 2005).

### Procedure and Participants

We started the adaptation of the Family Resilience Assessment Scale after obtaining permission from the author of the tool (Sixbey 2005). Similarly, to the process of adaptations in other countries (Kaya and Neslihan 2012; Bostan 2014; Dimech 2014; Li et al. 2016; Isaacs et al. 2018) the English version of the scale was first translated into Polish by a psychologist with a graduate degree in English, and two psychologists who are fluent in English and back into English by three other independent translators. The study was carried out using two participant samples. Firstly, a group of 26 students (84.6% women, M = 24.92, SD = 2.42) filled out the preliminary version of the questionnaire. This stage of research allowed for a correction of items that the respondents perceived as unclear. Then, after replacing the ambiguous words in certain items, a unified version was created. Next, three independent translators back-translated the items into English and this English version was compared to the original version of the Family Resilience Assessment Scale. The Polish version back-translated into English had a few differences, compared to the original, but they resulted from the need to adapt the vocabulary and concepts used in the Polish culture while keeping the meaning of the item, for example, the concept of the „Supreme Being” was replaced by the concept of „God” (item 3), „church/ synagogue/ mosque services” was replaced by „services” (item 12), „neighbors” was replaced by „people around you” (items: 11, 43) and the concept of,,community” was replaced by,,surroundings” (items: 19, 31, 32, 38, 49, 50). Terms „God”, „services”, „people around you”, “surroundings” were introduced because they are commonly used in Poland regardless of religion or place. The final version of the scale was approved by Sixbey.

Then, the second sample of participants filled out the Polish version of the *Family Resilience Assessment Scale* (Polish version name: *Skala Prężności Rodzinnej* FRAS-PL). This group consisted of 502 participants (65% women), between 18 and 62 years (M = 24.60, SD = 6.86), living in Poland in Pomerania. Most people were in their early adulthood (91%). The majority of the participants n = 323 (64%) had secondary education, followed by n = 170 (34%) who had higher education, and the rest (2%) had vocational qualifications. The majority of the participants (58%) were in a relationship (41% cohabiting, 17% married). In order to obtain the best-fitting model of a tool we used confirmatory factor analysis (CFA) with WLSMv estimator. The six-factor model was tested, which was then compared with the hierarchical model with the overarching factor of family resilience. The following indicators of goodness of fit were used: chi^2^, RMSEA, CFI, TLI, WRMR. Questionnaire items were analyzed in terms of statistical significance and their value. Next, we analyzed the reliability of the subscales, using Cronbach’s alpha. We used Mplus and IBM SPSS Statistics to conduct statistical analysis. The University of Gdańsk and the Medical University of Gdańsk in Poland approved this study.

## Results

Results of statistical analysis of six factor model, showing that the model fits data well: χ^2^ = 4015.941, df = 1362, χ^2^/df = 2.95, RMSEA = 0.06, CFI = 0.92, TLI = 0.91, WRMR = 1.87. The results of the statistical analysis do not confirm the original hierarchical model with the overarching factor (χ2 Test for the difference between the hierarchical and the six-factor model was 50.737, df = 9, p = 0.000). The result of a statistically significant χ2 test for testing the difference between the models indicates that the model of six identified factors better fits the data. Factor loadings of the items included in each of the subscales were statistically significant (p < 0.001) and higher than 0.30, therefore we decided to keep all of the items. The values of the factor loadings for the subscales are from 0.55 to 0.83 for *Family Communication and Problem Solving* (FCPS), from 0.32 to 0.81 for *Utilizing Social and Economic Resources* (USER), from 0.67 to 0.90 for *Maintaining a Positive Outlook* (MPO), from 0.40 to 0.91 for *Family Connectedness* (FC), from 0.64 to 0.97 for *Family Spirituality* (FS) and from 0.61 to 0.93 for *Ability to Make Meaning of Adversity* (AMMA). Table 1 shows factor loadings for the 54 items from the Polish version of FRAS-PL.

**Table 1:** Factor loadings for the FRAS-PL

Factors and items	Factor loadings	p
*Factor 1. Family communication and problem solving (FCPS)*		
1.Our family structure is flexible to deal with the unexpected	0.59	< 0.001
6. We all have input into major family decisions	0.68	< 0.001
7. We are able to work through pain and come to an understanding	0.69	< 0.001
8. We are adaptable to demands placed on us as a family	0.70	< 0.001
9. We are open to new ways of doing things in our family	0.66	< 0.001
10. We are understood by other family members	0.78	< 0.001
14. We can ask for clarification if we do not understand each other	0.80	< 0.001
15. We can be honest and direct with each other in our family	0.76	< 0.001
16. We can blow off steam at home without upsetting someone	0.56	< 0.001
17. We can compromise when problems come up	0.74	< 0.001
18. We can deal with family differences in accepting a loss	0.61	< 0.001
20. We can question the meaning behind messages in our family	0.77	< 0.001
23. We can talk about the way we communicate in our family	0.81	< 0.001
24. We can work through difficulties as a family	0.83	< 0.001
25. We consult with each other about decisions	0.75	< 0.001
26. We define problems positively to solve them	0.67	< 0.001
27. We discuss problems and feel good about the solutions	0.79	< 0.001
28. We discuss things until we reach a resolution	0.73	< 0.001
29. We feel free to express our opinions	0.74	< 0.001
30. We feel good giving time and energy to our family	0.73	< 0.001
40. We learn from each other’s mistakes	0.62	< 0.001
41. We mean what we say to each other in our family	0.69	< 0.001
46. We share responsibility in the family	0.69	< 0.001
48. We tell each other how much we care for one another	0.70	< 0.001
52. We try new ways of working with problems	0.55	< 0.001
53. We understand communication from other family members	0.72	< 0.001
54. We work to make sure family members are not emotionally or physically hurt	0.61	< 0.001
*Factor 2. Utilizing social and economic resources (USER)*		
11. We ask neighbors for help and assistance	0.33	< 0.001
19. We can depend upon people in this community	0.76	< 0.001
31. We feel people in this community are willing to help in an emergency	0.78	< 0.001
32. We feel secure living in this community	0.80	< 0.001
38. We know there is community help if there is trouble	0.81	< 0.001
39. We know we are important to our friends	0.72	< 0.001
43. We receive gifts and favors from neighbors	0.32	< 0.001
49. We think this is a good community to raise children	0.78	< 0.001
*Factor 3. Maintaining a positive outlook (MPO)*		
13. We believe we can handle our problems	0.70	< 0.001
21. We can solve major problems	0.88	< 0.001
22. We can survive if another problem comes up	0.90	< 0.001
34. We feel we are strong in facing big problems	0.67	< 0.001
36. We have the strength to solve our problems	0.73	< 0.001
51. We trust things will work out even in difficult times	0.64	< 0.001
*Factor 4. Family connectedness (FC)*		
2. Our friends value us and who we are	0.59	< 0.001
33. We feel taken for granted by family members	0.68	< 0.001
37. We keep our feelings to ourselves	0.73	< 0.001
45. We seldom listen to family members’ concerns or problems	0.65	< 0.001
47. We show love and affection for family members	0.91	< 0.001
50. We think we should not get too involved with people in this community	0.40	< 0.001
*Factor 5. Family spirituality (FS)*		
12. We attend church/synagogue/mosque services	0.92	< 0.001
35. We have faith in a supreme being	0.83	< 0.001
42. We participate in church activities	0.97	< 0.001
44. We seek advice from religious advisors	0.64	< 0.001
*Factor 6. Ability to make meaning of adversity (AMMA)*		
3. The things we do for each other make us feel a part of the family	0.93	< 0.001
4. We accept stressful events as a part of life	0.62	< 0.001
5. We accept that problems occur unexpectedly	0.61	< 0.001

The internal reliability of the subscales was satisfactory—for the five subscales the values of Cronbach's alpha are over 0.7. Only for one of the subscales the value is less than 0.7. Cronbach’s alpha values for the Polish version of the FRAS-PL are presented in Table 2.

**Table 2:** Cronbach’s Alpha for the Polish version of FRAS-PL

	Cronbacha’s Alpha FRAS-PL
Family communication and problem solving (FCPS)	0.95
Utilizing social and economic resources (USER)	0.78
Maintaining a positive outlook (MPO)	0.83
Family connectedness (FC)	0.77
Family spirituality (FS)	0.87
Ability to make meaning of adversity (AMMA)	0.63

Statistically significant correlations range from low for the Family Spiritual scale with the remaining subscales, through moderate and strong correlations of the other scales with each other. Table 3 shows the correlations between FRAS-PL subscales.

**Table 3:** Correlations between FRAS-PL subscales (CFA, WLSMv estimator)

	FCPS	USER	MPO	FC	FS	AMMA
FCPS	1.00					
USER	0.60***	1.00				
MPO	0.88***	0.56***	1.00			
FC	0.86***	0.65***	0.75***	1.00		
FS	0.13*	0.20***	0.12*	0.14**	1.00	
AMMA	0.74***	0.49 ***	0.75***	0.65***	0.08	1.00

*FCPS* Family communication and problem solving, *USER* utilizing social and economic resources, *MPO* maintaining a positive outlook, *FC* family connectedness, *FS* family spirituality, *AMMA* ability to make meaning of adversity

^*^ p < 0.05; ** p < 0.01; *** p < 0.001

## Discussion

The aim of the study was to adapt the *Family Resilience Assessment Scale* (FRAS) to the Polish language and culture and to examine its psychometric properties. The Polish version of FRAS was named Skala Prężności Rodzinnej (FRAS-PL). Family resilience depends on the family's life cycle, the history of difficult family events, family's ways of dealing with problems, or the type of problems encountered by the family. Family resilience also depends on the socio-cultural circumstances, which give a broader context to the family life (Bronfenbrenner 1979; Walsh 2006). This is why, the construct of family resilience can differ slightly in different cultural contexts. Previous FRAS adaptations conducted in Romania (Bostan 2014), Malta (Dimech 2014), Croatia (Ferić et al. 2016), and South Africa (Isaacs et al. 2018) yield a six-factor solution. Four factor solution was accepted in China (Li et al. 2016), and three factor in Turkey (Kaya and Neslihan 2012). Family Communications and Problem Solving (FCPS) and Maintaining a Positive Outlook (MPO) subscales have been accepted in China, Romania, Turkey, Malta, and Poland. This can mean that these two subscales are the least culturally dependent. The remaining scales: Utilizing Social and Economic Resources (USER), Family Connectedness (FC), Family Spirituality (FS), and Ability to Make Meaning of Adversity (AMMA) might be more influenced by culture, community and environment.

The original version of the Family Resilience Assessment Scale was created to evaluate family resilience among the population of the United States (Sixbey 2005). In the Polish version of the scale minor changes were made in the translation of meanings of some concepts (e.g. „community”, „neighbors”). This is due to the cultural differences between the North American and Polish people, but still provide a similar conceptual meaning. The confirmatory factor analysis (CFA) do not confirm the model with the general factor. The separated six-factor solution matches the data well and corresponds to the scales of the original FRAS model, leaving all positions. Although the p value for chi^2^ is significant (p < 0.001) and the WRMR value is greater than 1, we consider a six-factor solution acceptable, because other indices of goodness of fit (RMSEA, CFI, TLI) are good and the factor loadings for all 54 items are statistically significant and greater than 0.3.

Cronbach’s alpha values for the English subscales are between 0.70 for Family Connectedness and 0.96 for Family Communication and Problem Solving. In the Polish adaptation, the internal reliability scores for the five scales are lower than in the US version and range from 0.63 for the Ability to Make Meaning of Adversity to 0.95 for the Family Communication and Problem Solving. Only the Family Connectedness subscale obtains a higher Cronbach’s alpha value than the original version of this subscale (0.77 vs. 0.70). It should be noted that in the Polish version, only the Ability to Make Meaning of Adversity (AMMA) is lower than 0.70.

The correlations between subscales of FRAS-PL are consistent with Walsh’s (2006) assumption of interaction and synergy between family resilience processes. The strongest correlations are obtained between (1) Family Communication and Problem Solving (FCPS) and Maintaining Positive Outlook (MPO, r = 0.88, p < 0.001) and (2) Family Communication and Problem Solving (FCPS) and Family Connectedness (FC, r = 0.86, p < 0.001). Correlation is not obtained only between the Family Spirituality (FS) subscale and the Ability to Make Meaning of Adversity subscales (AMMA).

The research presented in this article focused on the process of adaptation of the English version of Family Resilience Assessment Scale (FRAS) to Polish culture. We note certain limitations of our study. First of all, the participants were mostly people in early adulthood, with higher and secondary education, living in the same area (Pomerania). Secondly, we did not study whole families but their individual members. Most importantly, we showed that the Family Resilience Assessment Scale (FRAS-PL) adapted well to the Polish culture, and it is a good tool for assessing family resilience. In the Polish scale adaptation, we have accepted a six-factor solution which is consistent with Sixbey’s (2005) scale and adaptations in Romania (Bostan 2014), Malta (Dimech 2014), Croatia (Ferić et al. 2016) and South Africa (Isaacs et al. 2018).

The values of the indexes of goodness of fit are good, and the internal consistency for the subscales is satisfactory, and the connection between subscales prove to be statistically significant. The obtained values of factor loadings for each item in all of the subscales are statistically significant and appropriate. The Polish version of FRAS-PL can be used by researchers and clinicians working with adults. We recommend calculating results for six subscales of family resilience. The next step will be to carry out a study to verify the criterion validity of the Polish version of the tool. To sum up, the Family Resilience Assessment Scale (Walsh 2005) based on model of family resilience processes created by Walsh (1996, 2003, 2006, 2013) can be applied to the Polish population.

The presented research is not free from limitations. Firstly, the study was conducted using self-report information only from one member of the family. In future research exploring psychometric qualities of FRAS-PL, it should be supplemented by information from both qualitative and other quantitative techniques. It would be also interesting to include in the research project whole families to check the differences in the perspective of family resilience of all the family members. Secondly, the study group has some specific demographic characteristics. Although the range of age of participants was from 18 to 62 years, most of them were young adults. Besides, the vast majority of participants had secondary or higher education. Therefore, the results may not easily be generalized to the whole Polish population. Additional research using FRAS-PL with other groups is needed. Despite the limitations, the study has its important contribution to the literature as it is the first tool in Poland which enables to conduct research on family resilience.
